# Assessment of laparoscopic skills of Gynecology and Obstetrics residents after a training program

**DOI:** 10.1590/S1679-45082016AO3752

**Published:** 2016

**Authors:** Carla Ferreira Kikuchi Fernandes, José Maria Cordeiro Ruano, Lea Mina Kati, Alberto Sinhiti Noguti, Manoel João Batista Castello Girão, Marair Gracio Ferreira Sartori

**Affiliations:** 1 Escola Paulista de Medicina, Universidade Federal de São Paulo, São Paulo, SP, Brazil.

**Keywords:** Education, medical, Laparoscopy, Internship and residency, Gynecology, Training, Surveys and questionnaires, Obstetrics and Gynecology department, hospital/manpower

## Abstract

**Objective:**

To evaluate laparoscopic skills of third-year Gynecology and Obstetrics residents after training at a training and surgical experimentation center.

**Methods:**

Use of a prospective questionnaire analyzing demographic data, medical residency, skills, competences, and training in a box trainer and in pigs.

**Results:**

After the training, there was significant improvement in laparoscopic skills according to the residents (before 1.3/after 2.7; p=0.000) and preceptors (before 2.1/after 4.8; p=0.000). There was also significant improvement in the feeling of competence in surgeries with level 1 and 2 of difficulty. All residents approved the training.

**Conclusion:**

The training was distributed into 12 hours in the box trainer and 20 hours in animals, and led to better laparoscopic skills and a feeling of more surgical competence in laparoscopic surgery levels 1 and 2.

## RESUMO

**Objetivo:**

Avaliar a habilidade laparoscópica dos residentes do terceiro ano de residência médica em Ginecologia e Obstetrícia após treinamento em um centro de treinamento e experimentação cirúrgica.

**Métodos:**

Aplicação de questionário de forma prospectiva analisando dados demográficos, da residência médica, da habilidade, da competência e do treinamento em caixa preta e em porcas.

**Resultados:**

Após o treinamento, houve melhora da habilidade em laparoscopia de forma significativa na avaliação dos residentes (antes 1,3/depois 2,7; p=0,000) e preceptores (antes 2,1/depois 4,8; p=0,000). Houve melhora significativa na sensação de competência em cirurgias de níveis 1 e 2 de dificuldade. Todos os residentes aprovaram o treinamento.

**Conclusão:**

O treinamento dividido em 12 horas de caixa preta e 20 horas em animais trouxe melhora na habilidade em laparoscopia e na sensação de melhora na competência cirúrgica em cirurgias laparoscópicas de níveis 1 e 2.

## INTRODUCTION

The patient consents to undergoing a surgical procedure based on the confidence deposited in his/her surgeon. This individual believes the surgeon is sufficiently experienced for the procedure, and considers him/her technically competent to entrust this task to their hands. In the doctor-patient, doctor-doctor interpersonal relationships, and even those among patients, a question arises: how to define surgical competence?. “What makes a surgeon competent?” Surgical competence is difficult to define.^(1)^

During decades, surgical training was based on the famous model “see, do, teach”, developed by William Halsted, in 1904. This model has produced a generation of good surgeons.^(2)^ Nevertheless, with the evolution of minimally invasive surgeries and with the appearance of new technologies, the acquisition of skills requires training outside the operating room to obtain a shorter learning curve and posteriorly, to apply the skills learned in the operating room.

Some studies showed that residents benefit from standardized teaching, and the combination of didactic classes with experience in animals improves surgical technique and skills.^(3-5)^ Today, educators in basic laparoscopy know that surgical skills need to be taught outside the operating room, and most trainings use varied methods, such as the box trainer, virtual simulators, animals, and cadavers.^(6-11)^ It is known that standardization, easy reproducibility of the exercises, immediate feedback after each training session and in the operating room, with an acceptable cost, bring benefits to teaching laparoscopy.^(12,13)^

Minimally invasive surgery became the objective of endoscopic surgeons throughout the world, and therefore, the teaching and training of endoscopy has become an issue of growing concern.

There is a large variety of models available for teaching, from animals and cadavers to simulators, each with its own advantages and disadvantages. The animal model has the advantage of being realistic, and the opportunity to mimic complications. However, it is criticized for the cost, anatomy, and ethical issues. Cadavers are rarely used due to the cost, limited availability, and impossibility of simulating complications. There are also bench models, which allow students to simulate different techniques, sutures, eye-hand coordination, and familiarity with the instruments. However, they require a specialist to demonstrate the procedure and to provide feedback about the performance. There are yet virtual simulators, which may replicate part of or the entire operation without the presence of a specialist, and furnish immediate feedback. These are criticized for their cost, limitation of tactile feedback, and lack of graphic realism.^(6,14,15)^

In the United States, in 2006, only 55% of residency programs had facilities for training in laparoscopy; in North America, in 2014, only 73% of programs taught laparoscopic skills.^(16,17)^ In Latin America, including Brazil, there is no teaching model for laparoscopic skills, or of validated tools for its evaluation. As a result, we believe that skill and expertise may vary among residents, depending on the type and number of cases they assisted. Thus, it is difficult to ensure that all residents will be exposed to the same procedures and will evaluate their skills.

Several authors have recently addressed this challenging question: “What makes a surgeon competent?” In asking this, we conclude that surgical competence is the product of many factors, including adequate medical knowledge, good decision-making regarding clinical, technical, and judgment issues; professionalism, interpersonal skills, communication with those involved, and technical knowledge. To teach skills in surgical technique is, therefore, one of the most important responsibilities.

In this way, in the present study, based on the evident need to improve teaching in surgical techniques for laparoscopy in Brazil, a training of third-year Gynecology and Obstetrics residents was proposed. Up until now, there are no published reports in Brazil on evaluations regarding improvement of laparoscopy skills in the laboratory. We believe that the training proposed may contribute towards teaching and training in laparoscopy for residents in Brazil.

## OBJECTIVE

To evaluate the laparoscopic skill of third-year Gynecology and Obstetrics residents after training at a surgical experimentation and training center.

## METHODS

In the present study, data were collected by means of questionnaires from 12 third-year Gynecology and Obstetrics residents of the *Escola Paulista de Medicina da Universidade Federal de São Paulo* (UNIFESP), from April 2011 to January 2012, evaluating the feeling of improvement in surgical skills after implementation of the teaching program at the *Centro de Experimentação e Treinamento em Cirurgia* (CETEC) [Center of Experimentation and Training in Surgery], at the *Hospital Israelita Albert Einstein.* All participants signed the Informed Consent Form, submitted to and approved by the Research Ethics Committee number 1263/11. Absence from CETEC at *Hospital Israelita Albert Einstein* for more than 15% of periods proposed, or poor completion of the questionnaire, would exclude the resident from the study.

The first questionnaire was filled out by the residents and contained data on demographics, education in laparoscopy during residency, interest in training with the box trainer and laboratory animals, interest, performance, and competence in laparoscopy, current laparoscopic skills, and factors that influence the application of laparoscopy in residency. It was applied to this group before and after training.

The second questionnaire was filled out by two preceptors on the first day and by the same preceptors on the last day of training.

Evaluation of the questionnaire was made by means of the Likert scale, expressing a 5-point scale, with 1 representing uncomfortable and 5, comfortable; 1 for no interest and 5 for great interest; or 1 for no importance and 5 for very important. The improvement parameter was considered as a 1.5 point increase in the Likert scale.

Training occurred during four hours weekly, for eight consecutive weeks, with three weeks destined to box trainer and bench training, and five weeks for surgeries in pigs, totaling up 32 hours of training assisted by the same preceptors with experience in advanced laparoscopic surgery.

The box trainer used measured 38,5cm by 29,5 on the base, and 20cm in height. A 10-mm 30 degrees camera was used, besides a power supply and an LCD monitor.

Training with a box trainer included eye-hand coordination exercises, and dissection and suture techniques. The exercises performed consisted in placing coffee into the cup, a match stick into the cup, dissecting gelatinous material and finding coffee beans inserted in it, forming a diamond shape with rubber bands on nails stuck into a wooden plaque, placing five pendants on a needle, and suturing and training of knots.

Different surgical interventions were performed with various degrees of difficulty, respectively: puncture with a Verres needle, creating pneumoperitoneum, passing trochanters, apprehension of pelvic structures with a Grasper clamp, lysis of adhesions, hysterectomy (corresponds to salpingectomy in humans), hysterectomy (corresponds to salpingectomy in humans), cholecystectomy, and nephrectomy. Care of animals complied with the norms of the Association for Assessment and Accreditation of Laboratory of Animal Care, and Normative Instruction number 7 of the Biosafety Technique Commission. The pigs used for surgery followed the rules of the *Sociedade Brasileira de Ciência em Animais de Laboratório* [Brazilian Society of Laboratory Animal Science].

The paired *t* test was used for comparison of results. The software used was Minitab, version 16.1 for data analysis.

## RESULTS

The 12 residents analyzed participated in all days of training. Only one questionnaire was excluded due to poor completion, with 11 questionnaires analyzed, seven of them from women (63.3%). The mean age was 28.2 years. Most residents had little experience in laparoscopy.

The residents were questioned as to their current skills in laparoscopy, which was the primary objective of the study (Table 1).

**Table 1:** Evaluation of current skill in laparoscopy before and after training

Likert Scale		p value (*t* test)

Before	After	
1.3	2.7	0.000

The residents observed between five to ten cases of laparoscopic surgeries and participated actively in zero to ten procedures. The cases observed by residents, and those where they had active participation, were considered insufficient for the learning process. The residents were also asked about the possible causes for their lack of skill during residency, and the main reason was the lack of training outside the operating room (Table 2).

**Table 2:** Evaluation of the possible causes of lack of skill during residency

Cause	Likert Scale 1-5 (mean)		

	Before	After	p value (*t* test)
Lack of training in the box trainer	4.9	4.7	0.341
Lack of training in a simulator	4.9	4.8	0.588
Lack of appropriate equipment	4.7	4.3	0.096
Not being an assistant surgeon	4.5	4.5	*
Not being the primary surgeon	4.1	4.8	0.136
Lack of cases	3.7	4.5	0.208
Lack of interest of the preceptor	3.5	3.7	0.714
Lack of interest of the resident	1.9	3.4	0.009

*Numerical data not applicable.

All residents had little prior experience in using the box trainer, and no experience in training with animals, and considered the training carried out very important (score above 4.5 on the Likert scale). Besides improving skill in laparoscopy, there was a statistical improvement in the perception of competence in performing level 1 and level 2 surgeries, and in level 3 myomectomies (probably due to the training with sutures), as shown on table 3.

**Table 3:** Evaluation of interest and competence in gynecological laparoscopy by levels of difficulty evaluated by the Likert scale

	Interest in laparoscopy			Self-perception of competence		
	
	Mean		p value (*t* test)	Mean		p value (*t* test)
	Before	After		Before	After	
Level 1						
Diagnostic laparoscopy	4.8	4.8	*	1.4	2.6	0.005
Tubal ligation	4.9	4.7	0.167	2.3	2.9	0.152
Level 2						
Ovarian biopsy	4.9	4.7	1.167	1.2	1.9	0.038
Lysis of adhesions	4.8	4.8	*	1.3	2.5	0.001
Ectopic gestation	4.9	4.8	0.341	1.1	2.2	0.001
Grades I and II Endometriosis	5.0	4.8	*	1.0	1.8	0.020
Oophoroplasty	5.0	4.9	*	1.1	1.8	0.024
Oophorectomy	4.9	4.8	0.341	1.1	2.1	0.004
Level 3						
Subtotal hysterectomy	4.8	4.5	0.104	1.0	1.4	0.104
Tubal reanastomosis	4.5	4.6	0.676	1.0	1.2	0.341
Myomectomy	4.9	4.6	0.277	1.0	1.5	0.053
Grades III and IV endometriosis	4.5	4.6	0.167	1.0	1.2	0.341
Sacropexy	3.8	4.0	0.441	1.0	1.3	0.192

*Numerical data not applicable.

### Analysis of the preceptor questionnaire data

Twelve residents were analyzed, but eight questionnaires were considered for the study. Three analyses were excluded, since one of the preceptors was not present on the day of the questionnaire, and one was excluded due to poor completion.

In the analysis, there was a significant improvement in the laparoscopic skill of the residents when evaluated by the preceptors (preceptor 1 and preceptor 2) (Table 4).

**Table 4:** Evaluation of laparoscopy skills of the residents performed by the preceptors

Likert Scale		p value (*t* test)

Before	After	
2.1	4.8	0.000

## DISCUSSION

Teaching surgical technique skills is one of the most important tasks in surgery training. Despite the importance of the inserting formal methods, few studies have been conducted in this area, especially in Gynecology and Obstetrics. Rapid advances in technology and surgical equipment increase the demand for time of the residents, and make teaching surgeons more challenging.

Several studies in the United States, Canada, France, and Netherlands have already established a formal program in the syllabus with some differences among them. In the same way, literature shows diversity in models of how to teach and evaluate surgical skills in Gynecology. In the present study, based on the evaluation of the training implemented, we have been able to observe an improvement of skills in laparoscopy, confirming that the program can be disseminated with the objective of training Gynecology and Obstetrics residents in Brazil; so far, there has been no training program in laparoscopy in Brazil.

Similar studies were performed in the United States and Canada, in 2002, with residents of Gynecology and General surgery, respectively, in which the Likert scale was applied. Gynecology residents exposed to a formal syllabus felt significantly more competent in performing several procedures as compared to those not exposed to the program. Most residents emphasized the need for training and its importance for their successful practice.^(18)^ General surgery residents had the expectation of performing basic and advanced procedures at the end of residency, and point out a lack of cases, lack of opportunity in the operating room, and a lack of support from the surgical department as factors that influenced their training in residency.^(19)^ The results of both studies were confirmed in the present study. The needs and expectations were similar, as well as the possible causes of difficulty in acquiring skills during residency.

In the literature, there still is no perfect model for teaching. The animal model proposed in the present training for gynecologists has shown efficacy in teaching. Exposing the residents to such diverse procedures, such as cholecystectomy and nephrectomy, with the intention of training the components used in non-gynecological procedures, albeit common in gynecological surgery, is beneficial. For example, in training the tactile sensation of dissecting tissues, the resident feels the difficulty of performing smooth and precise movements, in order to avoid lesions and possible bleeding, and if necessary, carry out immediate hemostasis. Moreover, learning to control force by means of the appropriate instrument, causing the least amount of damage to various tissues, such as bladder, uterus, Fallopian tubes, ovaries, intestinal loops, liver, gall bladder and kidneys. The performance of external or internal knots increases confidence and encourages the residents to consistently improve their technique.

## CONCLUSION

There was improvement in laparoscopic skills of Gynecology and Obstetrics residents after standardized training. We believe that other organizations can replicate the training described with the objective of enhancing the skills of residents and, consequently, the teaching of laparoscopy in Brazil.
